# Nature exposure might be the intervention to improve the self-regulation and skilled performance in mentally fatigue athletes: A narrative review and conceptual framework

**DOI:** 10.3389/fpsyg.2022.941299

**Published:** 2022-08-02

**Authors:** He Sun, Kim G. Soh, Samsilah Roslan, Mohd Rozilee Wazir Norjali Wazir, Alireza Mohammadi, Cong Ding, Zijian Zhao

**Affiliations:** ^1^School of Physical Education Institute (Main Campus), Zhengzhou University, Zhengzhou, China; ^2^Department of Sport Studies, Faculty of Education Studies, Universiti Putra Malaysia, Selangor, Malaysia; ^3^Department of Foundation of Education, Faculty of Education Studies, Universiti Putra Malaysia, Seri Kembangan, Selangor, Malaysia; ^4^Faculty of Business Management, City University Malaysia, Seri Kembangan, Selangor, Malaysia

**Keywords:** nature exposure, self-regulation, skilled performance, mental fatigue, intervention, conceptual framework

## Abstract

**Background:**

Due to causing inability of self-regulation (ego depletion) and executive functions such as directed attention and visual searching for relevant information (e.g., the ball location and the position of teammates), mental fatigue impairs skilled performance in various sports. On the other hand, natural scenes could improve directed attention, which may considerably benefit visual searching ability and self-regulation. However, nature exposure as a potential intervention to improve skilled performance among mentally fatigued athletes has not been discussed thoroughly.

**Purpose:**

To propose the potential intervention for the impairment of skilled performance among mentally fatigued athletes and generate a framework for future studies.

**Methods:**

A narrative review was applied to search broadly across disciplines, retrieving literature from several databases (PubMed, Web of Science, Scopus, and EBSCOhost).

**Results:**

Thirty-three works of literature including 39 experiments (mental fatigue 15; ego depletion 5; and nature exposure 19) were obtained. Finally, a conceptual framework was built regarding the effect of nature exposure intervention on skilled performance in athletes for future studies.

**Conclusion:**

Three theories (the psychobiological model of exercise performance, the strength model of self-regulation, and attention restoration theory) could be potentially integrated to be a conceptual framework and explain the mechanism of preventing prior mental exertion (e.g., mental fatigue and ego depletion). Future studies could examine more on the duration of the intervention.

## Introduction

Mental fatigue is a psychobiological condition resulting from extended, taxing cognitive activity (Hancock and Desmond, 2001), characteristically found among athletes in the matches. For a long time, the effect of mental fatigue on athletes’ performance only existed as an anecdote. For instance, Manchester team manager Roberto Mancini, in an interview with Eurosport TV channel, talked about worries that his side could fail to obtain silverware due to mental fatigue (Marcora, 2014).

Marcora (2014) found that mental fatigue decreased by 15% of the running distance in a Yo-Yo soccer-specific endurance test. The negative evidence came out strongly among athletes (e.g., soccer). The results were well proven by Grgic et al. (2022). Grgic et al. (2022) pooled the effect from nine studies and showed a negative effect of mental fatigue on the Yo-Yo test (Cohen’s *d*: −0.49; *p* < 0.001). Later on, numerous studies found that the reductions did not only occur in specific endurance performances but also in technique and decision-making (Badin et al., 2016; Smith et al., 2017; Fortes et al., 2019, 2020; Gantois et al., 2020). However, the intervention that can improve skilled performance (technical and decision-making skills) in mentally fatigued athletes remains limited. Notably, technical and decision-making skills play a key role in matches and they have been defined as skilled performance in sports, which is the capacity to perform at a high standard efficiently (Koopmann et al., 2020). In sports, It is also referred to as technical performance or skilled execution (Rampinini et al., 2007). The most recent review confirmed the adverse effect of mental fatigue on skilled performance (Sun et al., 2021), which could determine the outcome or result in sports (Allard and Burnett, 1985).

Rating perception of effort (RPE) plays a key role in endurance-based performance (Marcora et al., 2009; Van Cutsem et al., 2017). In contrast, it may not mediate the effect of mental fatigue on skilled performance. Recently, Habay et al. (2021) summarized all 21 literature investigated the sport-specific psychomotor performance and found that only two studies reported higher RPE (Badin et al., 2016; Veness et al., 2017). Unfortunately, these two studies (Badin et al., 2016; Veness et al., 2017) also involved endurance tests (e.g., walking and repeat sprints). Consistently, Soylu et al. (2021) examined all studies about the psychobiological response in soccer athletes. They found that only one study showed higher RPE in athletes after Yo-Yo IR1 test, rather than other skilled performance (e.g., technical and decision-making skills).

Notably, the execution of skilled performance is highly associated with athletes’ capacity for effortful attention (e.g., directed attention; Kaplan and Berman, 2010). That is, athletes in team sports (e.g., soccer) should perform reciprocally and sequentially, meaning that a phase of play in matches involves frequent moments of decision-making skills (Araújo et al., 2006; Bennett et al., 2019). Thus, the capacity to react fast (e.g., defending in a split-second) and accurately (e.g., passing the ball to a targeted teammate) is crucial among players. It requires players continually maintain concentration to perceive relevant information in the complex and rapidly changing environment (Afonso et al., 2012).

On the other hand, prolonged demand for a cognitive task that activates the anterior cingulate cortex (ACC), which in turn may cause feelings of mental fatigue due to reduced transmission of dopamine (MacDonald Angus et al., 2000; Lorist et al., 2005). Many studies argued that the impairment of skilled performance (e.g., technical and decision-making skills) is attributed to the reduction in transmission of dopamine, for it negatively influences executive function (e.g., attention) (Smith et al., 2018; McMorris, 2020; Sun et al., 2021). Consequently, mentally fatigued athletes cannot fully maintain directed attention to ignore irrelevant and distracting stimuli, and cannot think one step ahead and find the targeted teammate accurately, which is shown as an impairment in skilled performance. Thus, it is important to know how to counter these impairments, for athletes are bound to be exposed to mental fatigue during actual match (Smith et al., 2018; Russell et al., 2019). In a most recent study, Proost et al. (2022) confirmed the underlying mechanism with countermeasures. Authors argued that to combat mental fatigue, a modulator (e.g., music and caffeine) for the pathway of dopamine and adenosine is necessary.

Considering athletic outcomes, self-regulation is the ability to adjust physiological and psychological states adaptively to a specific context (e.g., soccer) (Baumeister and Vohs, 2003; Nigg, 2017). The depletion condition of self-regulation called ego depletion also impairs the subsequent physical and cognitive performance in sports (Englert and Bertrams, 2012; Furley et al., 2013). Players in team sports (e.g., soccer, basketball, Australian football, etc.) should exert self-regulation to continually maintain concentration to perceive relevant information in the complex and rapidly changing environment (Englert and Bertrams, 2012; Furley et al., 2013). However, it is perceived as effortful to self-regulate and consistently leads to symptoms of mental fatigue (Inzlicht et al., 2015). Subsequently, a reduction in skilled performance appeared among athletes. Notably, training self-regulation may provide another way to tackle mental fatigue, as it will consume fewer internal resources (Proost et al., 2022).

Moreover, some studies highly suggested evaluating mental fatigue and ego depletion together, for they are induced by common cognitive tasks (e.g., Stroop task, flanker task, AX-CPT) involving same executive function (e.g., inhibition), and both conditions are involved to the brain region of ACC (Giboin and Wolff, 2019; Brown et al., 2020). Furthermore, Brown et al. (2020) argued that separating the investigation of mental fatigue and ego depletion is the “biggest limitation.” This suggestion is also corroborated by a recent study by Graham and Brown (2021). The authors provided an in-depth overview of the effect of prior mental exertion (mental fatigue and ego depletion) on self-regulation of subsequent sports performance. Thus, the current review links these two independent research fields (mental fatigue and ego depletion) and tries to evaluate deep insight from theories, providing suggestions for future studies.

Nature exposure is a “direct physical or sensory contact with the natural environment” (Kamitsis and Francis, 2013). The relevant intervention could be a type of psychological therapy without any physical exercise and could be a technique (visualization or imagery training) to make people immerse in the natural settings (e.g., scenes) (Sun et al., 2022a). Many studies show that individuals can benefit in various ways from being exposed to nature, such as an enhancement in well-being (Grinde and Patil, 2009; Howell et al., 2011) and release of pressure (Pilotti et al., 2015). Most impor tantly, evidence demonstrates that nature exposure could restore directed attention (e.g., Berto, 2005; Beute and de Kort, 2014; Chow and Lau, 2015; Li and Sullivan, 2016), which may make mentally fatigued athletes concentrate back on the restoration of directed attention to retrieve relevant information and block out irrelevant stimuli (e.g., worrisome, cluster) in the competitive setting. Subsequently, the execution of skilled performance may be improved.

Furthermore, this benefit from nature scenes is undeniable and always happens, for humans are always inclined to affiliate with nature, which is called biophilia, supported by several substantial investigations (Joye, 2007; Howell et al., 2011; Berto et al., 2018). Also, nature exposure might enhance dopamine transportation in ACC (Darna et al., 2015), which could lead to restored directed attention and self-regulatory ability (Kaplan and Berman, 2010).

Sun et al. (2022a) did a promising study and examined the nature exposure intervention and showed it could considerably improve decision-making skills among mentally fatigued soccer players. Moreover, the best duration of the intervention was examined as 12.50 min, when the nature stimuli (e.g., nature scenes) were fixed. However, there is no conceptual framework to guide and show clear picture about why there was an improvement. More to the point, directed attention plays a key role in skilled performance, and it could be restored with nature stimuli (e.g., scenes) in a mentally fatigued or ego depletion population. Therefore, a new model or a conceptual framework is needed to comprehensively connect these factors and give suggestions to future studies.

**FIGURE 1:**
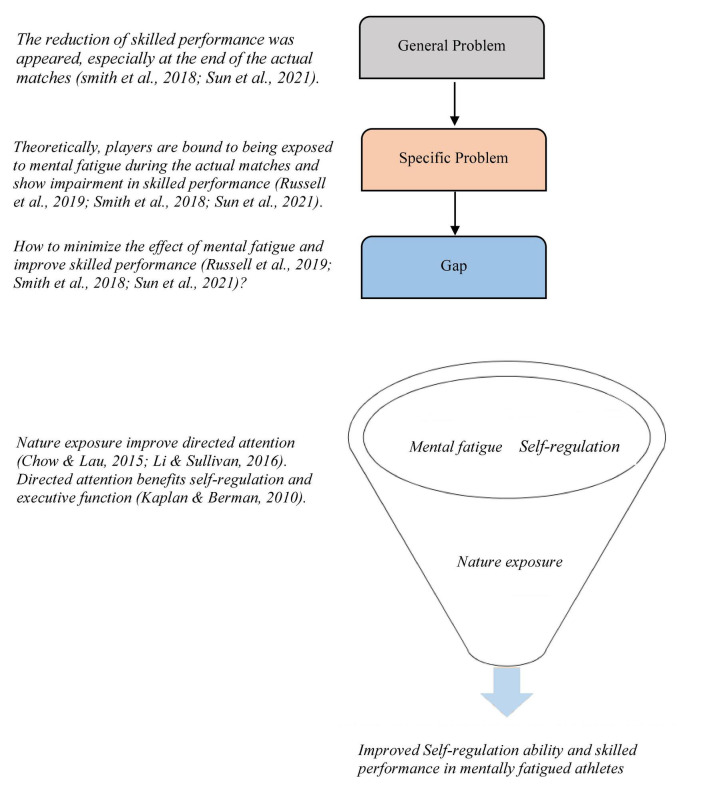
The study problem statement.

Since a narrative review could be an important scholarly tool to support theoretical explanations and discussions and unlike a systematic review, it enables searching more broadly across disciplines (Green et al., 2006; Jahan et al., 2016), the current study is a conceptual analysis based on a narrative literature review in three academic fields (mental fatigue, ego depletion, and nature exposure academic fields). It aims to build a conceptual framework that might significantly counter mental fatigue and improve the subsequent skilled performance. Figure 1 shows the problem statement identified in the current review.

## Methodology

Following the methodological framework to write a narrative review (Green et al., 2006), we conducted the current review with four steps; they are: identifying sources, defining the searching strategy and setting parameters, defining selection criteria, and summarizing and synthesizing results.

### Identifying sources

A literature search was conducted employing several databases, including PubMed, Web of Science, Scopus, and EBSCOhost (CENTRAL, SPORTDicus) from the inception of databases to March 2022. Moreover, we also manually searched Google Scholar for gray literature that could have evaded our search parameters in these databases due to different indexing.

### Defining the searching strategy

To comprehensive boundaries for the literature, two series of search terms were used: (i) “mental fatigue” OR “mental exertion” OR “cognitive fatigue” OR “cognitive exertion” OR “mental exhaustion” OR “mental tiredness” OR “ego depletion” AND “athletic performance” OR “technical skill*” OR “skill*” OR “technique” OR “decision making” OR “performance” AND sport*; and (ii) “mental fatigue” OR “mental exertion” OR “cognitive fatigue” OR “cognitive exertion” OR “mental exhaustion” OR “mental tiredness” OR “ego depletion” AND Nature*. The results of the searching show in Supplementary Tables 1, 2.

### Defining selection criteria

To identify the relevant literature, several steps were done separately by two series of search terms (Supplementary Figure 1); they are (i) yielded a total number of the literature searching in databases and Google Scholar; (ii) using endnote and removing duplicates; (iii) title and abstract screening to identify whether relate to ‘skilled performance’; or ‘nature exposure’ in mental fatigue or ego depletion academic field; (iv) full-text review proceeded. Moreover, any investigation conducted without prior mental exertion task to induce fatigue and with unhealthy subjects was excluded. Two reviewers independently evaluated the inclusion of these works. Any disagreement was solved by consensus or a third reviewer discussion.

### Summary and synthesis

Excel was used to design a data extraction sheet to compile a summary of the research topic based on the included literature. And synthesized the purpose of each study, methods or material utilized, variables investigated, any findings related to ‘skilled performance’ and ‘nature exposure,’ and comments or note from authors. The relevant findings finally were presented in the following section.

## Results

Thirty-three works of literature including thirty-seven experiments (mental fatigue 15; ego depletion 5; and nature exposure 19) were obtained. The details showed in Tables 1–3, respectively.

**TABLE 1:** Summary studies of knowledge about the effect of mental fatigue on skilled performance in sports.

Publication	Subject	*N*	Prior cognitive task	Duration (min)	Sport-skill task	Outcome
Badin et al., 2016	Elite	20	Stroop task Computer version	30	Soccer: small-sided games	MF↑ RPE↑ MO↔ Passing accuracy%↓ Tackle success↓
Smith et al., 2016a	Well-trained	14	Stroop task Paper version	30	LSPT	MF↑ RPE↔ MO↔ Penalty time↑ Performance time↑ Points per shot↓ Shot speed↓
Smith et al., 2016b	Professional	12	Stroop task Paper version	30	Soccer: decision-making task	MF↑ MO↑ Overall response accuracy↓ Response time↑ Visual search↓
Smith et al., 2017	Well-trained	14	Stroop task Paper version	30	LSPT, LSST	MF↑ MO↑ Missed target↓
Le Mansec et al., 2017	Professional	22	AX-CPT	90	Table Tennis: forehand strokes	MF↑ RPE↔ Ball speed↓ Accuracy↓ Faults number↑ Total score↓
Moreira et al., 2018	Elite	48	Stroop task Computer version	30	Basketball: small-sided game	MF↑ RPE↔ Salivary parameters↑ Alpha-amylase↑ Athletes’ efficiency↔ Turnover number↑
Fortes et al., 2019	Professional	20	Smartphone	30	Soccer: full-sided game	MF↑ RPE↔ Passing decision-making↓
				45		
Gantois et al., 2020	Professional	20	Stroop task Computer version	30	Soccer: full-sided game	MF↑ RPE↔ Passing decision-making↓
Fortes et al., 2020	Professional	25	Smartphone	30	Soccer: full-sided game	MF↑ Penalty↓ Performance time↓ MF↑ RPE↔ Lactate Blood↔ Passing decision-making
		25	Video games			
Trecroci et al., 2020	Sub-elite	9	Stroop task Smartphone app	30	Soccer: small-sided game	MF↑ RPE↔ Negative pass↑ Shot accuracy↓ Dribbling accuracy↓
Filipas et al., 2021a	Sub-elite	36	Stroop task Computer version	30	Soccer: LSPT, LSST	MF↑ Penalty↓ Performance time↓
Filipas et al., 2021b	Amateur	19	Tactical basketball video	30	Basketball: free-throw performance	MF↑ MO↔ Throw accuracy↓
		19	Sleep restriction	<5 h		
			Tactical basketball video + sleep restriction			
Fortes et al., 2021a	Regional level	30	Smartphone	30	Box skilled performance	MF↑ RPE↑ Decision-making index (offensive and defensive) ↓
		30	Video game			
Weerakkody et al., 2021	Amateur	25	Stroop task	35	Australian football	MF↑ Brad Johnson goalkicking accuracy↓
Bian et al., 2022	Well-trained	15	Stroop task	20	Soccer LSPT	MF↑ MO↔ Movement time ↔ Penalty time↑ Passing accuracy↓

LSPT, Loughborough Soccer Passing Test; LSST, Loughborough Soccer Shooting Test; MF, mental fatigue; MO, motivation; RPE, rating of perceived effort; ↑, higher; ↓, lower; ↔, no significant difference.

**TABLE 2:** Summary studies of knowledge about the effect of ego depletion on skilled performance in sports.

Publication	Subject	*N*	Prior cognitive task	Duration (min)	Sport-skill task	Theory	Outcome
Englert and Bertrams, 2012	Amateur	64	Transcription task	6 min	Basketball free throw	SMSR	Ego depletion↑ Throw performance lowered in the high anxiety and depleted group.
		79			Dart performance		
Furley et al., 2013	Semi-professional	40	Transcription task	6 min	Basketball decision-making task	SMSR	Ego depletion↑ Decision-making skill in the basketball representative task decreased in the depleted group.
Englert et al., 2015a	Semi-professional	57	Transcription task	6 min	Tennis serve task	SMSR	Ego depletion↑ Tennis serve performance decreased in the depleted group.
Englert et al., 2015b	Amateur	28	Transcription task	6 min	Dart throwing	SMSR; and Integrated Model	Ego depletion↑ Fixation duration was shorter in high state anxiety of depleted group.
Shaabani et al., 2020	Experienced	72	Stroop task	15 min	Basketball free throw	SMSR	Ego depletion↑ Throwing scores decreased in the depleted groups.

SMSR, the strength model of self-regulation; ↑, higher; ↓, lower; ↔, no significant difference.

**TABLE 3:** Summary studies of the effect of nature exposure on cognitive performance.

Publication	*N*	Prior cognitive task	Duration	Intervention description and specific method	Theory	Outcome
Laumann et al., 2003	25	Proofreading task	15 min	*Nature exposure:* nature surrounding restore depleted voluntary attention. **20 min** nature scenes video, depicting waterside environment.	ART	C: Posner’s attention-orienting task Valid trials for the exogenous ↑
Berto, 2005 Experiment 1	32	SART	5 min	*Nature exposure*: restorative environments facilitate recovery from mental fatigue. 25 restorative pictures, each picture was showed 15 s on the computer. **6.25 min**	ART	C: SART D-prime; reaction time and correction responses↑
Experiment 2				25 geometrical patterns, each pattern was showed 15 s on the computer. **6.25 min**		D-prime and reaction time incorrect responses↓
Experiment 3				25 restorative pictures, self-space exposure time, which was less than 15 s for each picture. **Less than 6.25 min**		D-prime and correction response↑
Berman et al., 2008 Experiment 1	38	Directed-forgetting task	35 min	*Nature exposure:* interaction with nature restore directed attention. **50–55 min** walk in nature	ART	C: Backward digit-span Corrections↑
Experiment 2	18			50 nature pictures were showed 7 s of each. **5.83 min**		C: Backward digit-span Corrections↑ Attention network test: Executive functions↑ Alerting and orienting↓
Beute and de Kort, 2014 Experiment 1	90	Typing task	UA	*Nature exposure:* improve self-regulation and executive function. Subjects rated the presented slideshow about nature**. 3 min**: 20 s each, 9 pictures.	(1) ART (2) SMSR	C: Stroop task Reaction time↓ Errors↔
Experiment 2	121	Typing task and Stroop task	Stroop task 4 min			C: 2-back task Reaction time↔ Errors↔
Emfield and Neider, 2014	202	Cognitive battery task (3 types)	30 min	*Nature exposure:* nature scenes restore directed attention. **5.83 min**: 50 nature photos, 7 s each; nature sound exposure; nature pictures + sound.	ART	C: Backward digit span Remember words↔ Attention network task Corrections↔ FFOV Accuracy↔
Chow and Lau, 2015 Experiment 1	42	Transcription task	10 min	*Nature exposure:* (1) the self-regulatory strength can be replenished via rest; (2) rich in soft fascination helps people recover from mental fatigue and improve directed attention. Subjects were provided a picture-album that consisted of natural settings for **6 min.**	(1) ART (2) SMSR	C: Anagram task Persistence time↑
Experiment 2	58	Perception task	6 min	Subjects viewed nature scenes of slides. **4 min:** 4 pictures, each 1 min.		C: Logical reasoning task Logical scores↑
Experiment 3	185	Retyping task	6 min	Subjects were asked to view nine nature pictures and clicked on the area of the pictures that attracted their attention. **1.5 min:** 9 picture, 10 s each.		C: Anagram task Number of solved anagrams↑
Lee et al., 2015	150	SART	5 min	*Nature exposure:* nature in cities is restorative. Subjects self-determining length of viewing time; viewed a “green roof” planted with a meadow containing taller green grass and yellow flower. **40 s**	(1) ART (2) Attention-resource Model	C: SART Omission, errors, slow-frequency gradual response and fast-frequency moment-to moment response↓
Evensen et al., 2015	85	Computer work	1 h	*Nature exposure:* nature renew psychological resources that have been depleted; positive affect. Four plants as the interior; inanimate objects replicated plants. **1 h work with 5 min break.**	(1) ART (2) Stress Recovery Theory	C: Reading span task Correct words↑
Pilotti et al., 2015	63	A workday	1 day	*Nature exposure:* nature scenes are good at physiological indices, performance, and self-report measures of well-being. **15 min** video about nature.	ART	C: Sustained attention task Response latencies↔ Memory test Long-term memory↑
Haga et al., 2016	90	A cognitive demanding task	40 min	*Nature exposure:* natural settings have greater restorative effects on psychological resources recovery. **3 min** with sound about nature environment with a streaming waterfall.	ART	C: Attention network test Response-time↔ Accuracy↔
Zhang et al., 2017	70	Reasoning test	50 min	*Nature exposure*: nature promote the restoration of people from the state of direction attention fatigue. **40 min** nature sound (birds, water, etc.) in the real environment.	ART	C: Complement test Grade of complementary pairs↑
Bennett, 2019	116	Math problems task	15 min	*Nature exposure:* nature settings attract involuntary attention, restoring directed attention. Expose nature sound with headphones (bird songs). **4 min**	ART	C: Backward digit-span Corrections↔
Neilson et al., 2020	60	SART	5 min	*Nature exposure:* restorative environments facilitate recovery from mental fatigue. 25 restorative pictures, each picture was showed 15 s on the computer. **6.25 min**	ART	C: SART Reaction time and correction responses↔

ART, attention restoration theory; SMSR, the strength model of self-regulation; SART, Sustained Attention to Response Test; C, cognitive test; ↑, higher; ↓, lower; ↔, no significant difference.

### The effect of mental fatigue on skilled performance

As shown in Table 1, mental fatigue negatively influences skilled performance, including technical and decision-making skills in several sports (soccer, basketball, table tennis, boxing, and Australian football).

**FIGURE 2:**
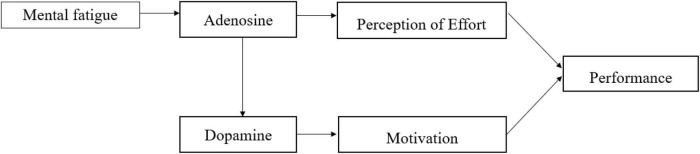
The representation of the psychobiological model. The representation of the model is from the study of Martin et al. (2018).

It is worthy to note that among the prominent theories of mental fatigue is the psychobiological model of exercise performance that was proposed by Marcora (2008). The model is based on motivational intensity theory (Brehm and Self, 1989), and provides an explanation for the adverse effects of mental fatigue on physical performance (reduced exhaustion time and output of self-selected strength). The model further highlighted two factors: motivation and RPE that determined the consciously regulated behavior. The model has also been applied to soccer, however, the same as the original model merely related to the physical domain, intermittent endurance showed impairment due to elevated RPE (Smith et al., 2015). Furthermore, Martin and colleagues indicated that mental fatigue alters the concentration of dopamine in the ACC and that raising exercise motivation or decreasing the RPE could counter this negative alteration (Martin et al., 2018; Figure 2).

However, the current review (Table 1) found inconsistent evidence, as there are only two studies that showed the level of RPE was significantly different in the mental fatigue group (Badin et al., 2016; Fortes et al., 2021a). That is, the reductions of skilled performance could not be attributed to RPE, including technical and decision-making skill.

Notably, Moreira et al. (2018) did a well-done study and examined the effect of mental fatigue on basketball performance. According to their findings, mental fatigue increases levels of salivary testosterone, which could explain why athletes make more mistakes in technical skills. To be sure, the dopamine-containing mesolimbic reward system is believed to be regulated by testosterone (Robbins and Everitt, 1996). Dopamine transmission may be affected by lower testosterone levels in mentally fatigued athletes, according to Moreira and colleagues. Mental fatigue reduces dopamine levels in the ACC, which impairs executive functions, as has been shown in previous studies (Smith et al., 2018; Sun et al., 2021).

Impairment of executive functions, such as attentional direction, has been linked to technical skill impairment (Badin et al., 2016; Smith et al., 2016a,^2017^). As a result of mental fatigue, attention can be diverted from goal-directed to irrelevant stimuli-directed processing (Boksem et al., 2005; Csathó et al., 2012). Furthermore, mental fatigue also diminished the ability to prepare to control the ball movement (Smith et al., 2017). The scenario could be more likely found in actual match due to the additional distracted stimuli (Smith et al., 2016a).

### The effect of ego depletion on skilled performance

When considering athletic outcomes, self-regulation is the capacity to adjust physiological and psychological states adaptively to the specific context (e.g., soccer) (Baumeister and Vohs, 2003; Nigg, 2017). Athletes must also be able to voluntarily decrease anxiety and maintain attention (Englert and Bertrams, 2012); in other words they must be capable of self-regulation (Baumeister and Vohs, 2003; Nigg, 2017). Consistently trying to self-regulate has shown to increase the risk of future self-regulation failure. Ego depletion refers to this psychological state (Baumeister et al., 2000).

Table 2 shows that ego depletion impairs skilled performance among athletes in the sports of basketball, tennis, and dart throwing. Importantly, these impairments have been attributed to the reduction of an executive function, which is directed attention (Englert and Bertrams, 2012; Furley et al., 2013; Englert et al., 2015b; Shaabani et al., 2020). For example, when making a tactical decision in team sports (e.g., basketball) while the opposing audience is calling an apparent rule violation, athletes should exert directed attention to block out irrelevant distractions and only concentrate on the relevant information. Also, dart athletes need to make longer fixations on the target and simultaneously suppress attention to task-irrelevant stimuli. Because all self-regulation processes use the same finite amount of brain resources, as proposed by the strength model (Baumeister and Heatherton, 1996; Baumeister et al., 1998), applying self-control throughout a cognitive task such as transcription task would have a negative impact on the ability to perform skilled task (Table 2) that unrelated self-regulation demanding. Therefore, to counter this negative effect, it is necessary to increase the athletes’ ability of attention to retrieval of relevant information.

Consistently, the current review confirmed the suggestion for the combined investigation of mental fatigue and ego depletion (Giboin and Wolff, 2019; Brown et al., 2020), for two fields usually recruited the same cognitive function to induce the certain condition. Table 2 shows the most common cognitive task which was used to induce the condition of ego depletion is 6 min transcription task. In the depletion group, athletes were usually asked to omit the letters “e” and “n.” For successful task completion, inhibition is inevitable to be exerted as one has to volitionally override one’s writing habits (Schmeichel, 2007). Similarity, inhibition is typically assessed with versions of the Stroop task (Stroop, 1935) in which participants have to override or inhibit a prepotent response to the different color of words used frequently to induce mental fatigue conditions (see Table 1).

### The effect of nature exposure on prior mental exertion

According to the results in Table 3, many studies showed nature exposure intervention restored directed attention after a particular mental exertion task. For example, Berto (2005) recruited 5 min Sustained Attention to Response Test to induce mental exertion. The result showed after 6.25 min nature exposure, the directed attention measured as reaction time and correct response were significantly improved. Consistently, Chow and Lau (2015) employed transcription, perception, and retyping tasks as the prior mental exertion. The result showed that nature exposure intervention improved directed attention dramatically measured in anagram and logical reasoning tasks.

Notably, Table 2 also shows some other studies did not improve directed attention significantly after the intervention. Probably because the ‘dosage’ or durations of nature exposure intervention used in all of these studies are different. Specifically, Laumann et al. (2003) employed a 20-min nature exposure and found significant results after the 15-min mental exertion exercise done by its participants, whereas Bennett (2019) used a 4-min intervention to counteract the effects of a 15-min mental exertion, but found no improvement. In addition to those studies, Beute and de Kort (2014) found that 3 min of nature exposure significantly increased cognitive performance after performing a typing task. In contrast, they did not find any improvement after adding a 4-min Stroop task to their prior mental exertion task with the same nature exposure intervention time. This ‘dosage’ or duration issue also was raised by a previous systematic review (see Stevenson et al., 2018).

Besides the duration, the type is also crucial for future studies to implement the intervention. Berman et al. (2008) particularly investigated two types (actual and virtual scenes) of nature exposure. In the first study, the authors recruited the actual setting and did a 50–55 min intervention. The result showed after 50–55 min walking in this actual nature environment, there was a significant restoration in directed attention. The improvement was also detected, when participants were exposed to the virtual scenes including some particular nature stimuli shown on the computer (see the detail of virtual nature in the following section: The Generation of a Conceptual Framework); however, the duration of the intervention is much shorter than actual setting (5.83 min vs. 50–55 min; Table 3). In fact, it is particularly difficult to maintain ecological validity in actual environments. To contrast, the ecological validity could be reconciled very well in virtual nature (e.g., virtual reality) with experimental control. Research into how to make a person feel more comfortable and fully immersed in a virtual environment has been extensive (Stone, 2008). The realism of a setting could be considered a loose analogy for the concept of immersion in this context (Brown and Cairns, 2004). Biophilia of humans is the key and responsible for positive cognitive responses, restoring directed attention capacities in nature (Joye, 2007; Howell et al., 2011; Berto et al., 2018). Across studies, it was typical for actual exposure to be longer than virtual exposures (Berman et al., 2008; Chow and Lau, 2015; Zhang et al., 2017; Neilson et al., 2020). Since people decreased outdoor activities with the COVID-19 situation (Freeman and Eykelbosh, 2020), virtual exposure could be considered more in future studies.

### Theories rational

The conceptual framework developed in this study was based on three theories, which are the most prestigious ones in mental fatigue, ego depletion, and nature exposure reach field, respectively; they are The pychobiological model of exercise performance (Marcora, 2008, 2009), The strength model of self-regulation (Baumeister and Heatherton, 1996), and attention restoration theory (ART) (Kaplan and Berman, 2010).

#### The psychobiological model of exercise performance

**FIGURE 3:**
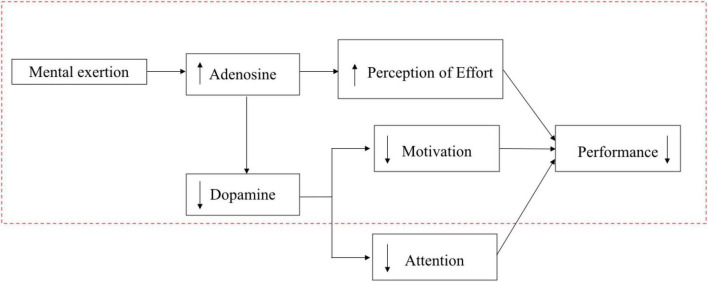
The potential update of the mechanism for psychobiological model of exercise performance. The schematic representation of the mechanism for the model (inside the dotted line) is from Martin et al. (2018).

The original psychobiological model of exercise performance mainly indicates the reason for physical performance is impaired by mental fatigue and has two factors, which shows in the dotted line of Figure 3 shown in the study of Martin et al. (2018).

Inconsistent with the model, only Badin et al. (2016) and Fortes et al. (2021a) reported a significantly different RPE between the experimental and control group (see Table 1: outcome). Other studies did not detect any difference between mental fatigue and the control group regarding RPE and motivation level (e.g., Smith et al., 2016a; Filipas et al., 2021b). Therefore, an updated model is required to apply the model to the domain of skilled performance.

Notably, the skilled performance was significantly impaired by a reduction in executive function, which may have been caused by mental fatigue tasks activating the ACC. In turn, it may have resulted in elevated adenosine and decreased dopamine level (Smith et al., 2018; Fortes et al., 2020). Especially, attention plays a key role in this impairment in terms of reduction of executive function.

Moreover, the shift in attention focus impairs athletes’ decision-making. Decision-making in soccer is heavily reliant on the human brain’s ability to perceive relevant information from a complex environment while filtering out irrelevant distractions (Baker et al., 2003; Gantois et al., 2020). Selective attention governs this process (Kastner et al., 1999; Murray and Wojciulik, 2004). Selectivity allows athletes to make flexible decisions by preventing them from reacting reflexively to environmental stimuli. However, it was hampered by mental fatigue (Smith et al., 2016b; Fortes et al., 2019, 2021b; Gantois et al., 2020). Directed attention refers to the ability to focus attention selectively and sustainably (Cohen, 2018).

Although the actions of dopamine in the brain are complex, research shows that the natural environment enhances dopamine transportation (Darna et al., 2015), restoring directed attention and improving the ability of self-regulation (Francis and Inzlicht, 2016). Also, because directed attention and executive function are reciprocal (Karyotaki et al., 2017), a third alternative is that replenishment of the capacity of directed attention through restorative environments may lead to reduced performance declines as mental fatigue is minimized or prevented. Consequently, the psychobiological model may be expanded (see Figure 3, outside the dotted line).

#### The strength model of self-regulation

There are two gist in the strength model of self-regulation: (i) global and (ii) finite.

(i) Global

The model has proposed that self-regulation is universal global strength that enables people to perform tasks (Hagger et al., 2010). Baumeister and Vohs (2003) emphasized that self-regulation is a common resource for responding to diverse tasks and functions. A body of research exemplified this by showing how one form of response impairs the subsequent control over other various forms of responses. Even the common prefrontal region in the brain is activated for different types of tasks that each require resources. Researchers further concluded that any act of self-control should tax one’s strength, and that afterward, one is less ability of self-regulation. A fluctuating capacity in any subject means that self-regulation is costly in short term, which would underpin individual variation in performances requiring self-regulation.

(ii) Finite

Another major tenet of this theory is that the capacity of self-regulation is a finite “reservoir.” The resource is recognized as limited, to be depleted over time. Originally, Baumeister and Heatherton (1996) and Baumeister et al. (1998) indicated that the strength is the same as that of the muscle, which requires energy to perform and becomes fatigued over a period of consecutive exertion. This means that the exertion of self-regulation can only be maintained for a limited period, and the resource is prone to depletion over time. Similarly, it can also be recuperated after a period of rest.

Therefore, after the first cognitive task (e.g., Stroop task), the self-regulation resources were depleted, induced condition ego depletion or mental fatigue, and subsequent performance was impaired.

#### Attention restoration theory

Attention restoration theory comes from the research field of environmental psychology. It proposes that nature exposure can restore directed attention. Specifically, James (1892, as cited in Kaplan and Berman, 2010) suggested that voluntary effort to resist temptation and behave oneself is typical of self-regulation and the executive function, due to the centrality of attention in both. As he briefly defined it, “voluntary effort is the effort of attention” (James, 1892, p. 317, as cited in Kaplan and Berman, 2010). James further believed that the nebulous concept of resources and effort is made up of more concrete processes (e.g., attention), which can help us to define more precisely the finite resource.

In addition, James (1892, as cited in Kaplan and Berman, 2010) identified two forms of attention, differentiated by the amount of effort it takes to use each one. when something interesting or exciting happens, such as the appearance of a wild animal, it is so-called involuntary attention. Another form of attention was elicited by these interesting stimuli, which could be interpreted as an act of “seeking to discover what is going on.” For example, a color snake in the wild is witnessed by a young child who has never seen it before. Such a scene may cause everything else in the world to disappear (Larson and Memtt, 1991). Generally, environmental features capture this attention. However, it needs to be sufficiently gentle to be far away from other interferences and is referred to as ‘soft fascination’ according to ART, which largely exists in the natural environments (Kaplan and Berman, 2010).

On the other hand, the counterpart of attention, the so-called voluntary attention or directed attention requires a lot more effort (Morecraft et al., 2015), forces oneself to focus on something uninteresting or tedious. A study on the mechanism found that voluntary attention is driven more by the prefrontal cortex neurons (Buschman and Miller, 2007), which is the same for self-regulation (Baumeister and Vohs, 2004), whereas involuntary attention is driven more by other parts of the brain.

Based on James’ identification of attention, Kaplan, 1995, 2001 proposed ART and suggested that voluntary attention is effortful and can be tiring, whereas involuntary attention is effortless and helps the attention system to rest and restore. Kaplan and Berman (2010) further argued that directed attention shares a common resource with self-regulation. The hypothesis was tested by some studies. For example, Naghavi et al. (2018) demonstrated that cognitive training could improve the capacity of self-regulation measured as psychophysiological indicators (e.g., HRV and skin conductance). Similarly, the attentional control theory and the strength model of self-regulation were integrated by Englert and Bertrams (2015). The authors indicated that under high levels of anxiety, attention regulation is more susceptible to distraction. Players could focus on the relevant target while blocking out distracting stimuli. Therefore, they could maintain their sports performance.

To summarize, there are potential connections among these three theories (the psychobiological model of exercise performance, the strength model of self-regulation, and attention restoration theory). It may be fruitful to integrate them and provides a guide to future studies to improve the skilled performance among mentally fatigued sports players. However, a conceptual framework is needed.

### The generation of a conceptual framework

**FIGURE 4:**
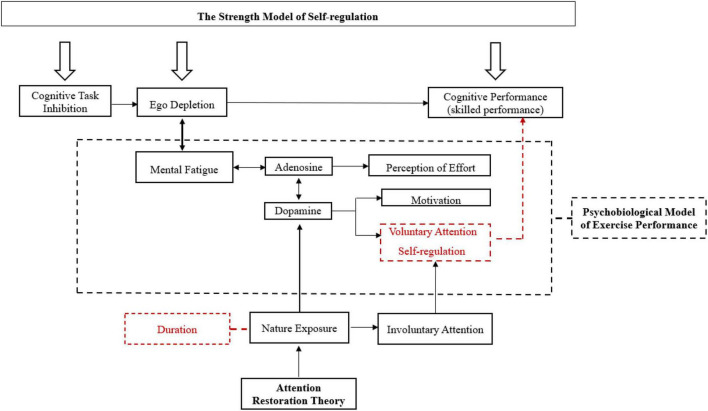
A conceptual framework for countering mental fatigue and improving skilled performance.

According to an in-depth literature review, a conceptual framework was generated as shown in Figure 4 with three theories regarding the nature exposure intervention.

Specifically, the strength model of self-regulation indicates that reduced performance in the second cognitive task (e.g., skilled performance) appears after the first prolonged cognitive task since the self-regulation resources are limited. The condition of depleted resources is ego depletion or mental fatigue.

Moreover, ART indicates that nature exposure effectively attracts involuntary attention, leading to restoring voluntary attention (directed attention). This process is relevant to the increase of dopamine in ACC (Darna et al., 2015). The mentally fatigued players may have better self-regulation ability, and subsequent decision-making may be also improved after nature exposure intervention since directed attention is overlapped with self-regulation (Kaplan and Berman, 2010).

Directed attention may be the third factor to prevent reduced performance declines as mental fatigue is minimized, for directed attention and executive functions are reciprocal (Karyotaki et al., 2017), and dopamine transportation is enhanced (Darna et al., 2015) in the Psychobiological Model of Exercise Performance.

## Discussion

The current review built a conceptual framework through a thorough literature review and gave suggestions for future studies. Eventually, the review associated nature exposure with skilled performance and showed potential integration according to three theories.

This framework further helps athletes and coaches to understand the mechanism of preventing prior mental exertion (e.g., mental fatigue and ego depletion) based on some variables, such as the duration of nature exposure, self-regulation and directed attention shown in Figure 4.

Notably, nature exposure intervention should be implemented based on the restorative environment, which is full of “soft fascination” (Kaplan and Berman, 2010). This soft fascination is essential, for it softly attracts involuntary attention while at the same time limiting the need for directed attention. It is suitable to implement this intervention virtually, rather than in the real environment. There are mainly two reasons: (i) Stevenson et al. (2018) argued that to achieve the restorativeness, exposure to a virtual environment can be shorter than a real environment, probably because it is too difficult to control distractions (e.g., artificial scenes or sound) in the real environment; (ii) People greatly decrease the outdoor exposure time since COVID-19 outburst (Pancani et al., 2021; Wright et al., 2021).

However, compared to natural stimuli, such as images, sound, though a part of a natural setting, has fewer positive effects (Haga et al., 2016). Thus, to make people fully immersed, future studies should fully consider the criteria selection of nature scenes. Nature scenes, according to Balling and Falk (1982), are those in which there are no artifacts, but there is no evidence of human management, as in national forests or national parks. Hence, the domain of nature is by no means limited only to the wilderness (Ulrich, 1983). From a broad perspective, Ulrich (1983) and Han (2003) indicated that nature scenes should be fulfilling three conditions: (a) the dominant contours are curvilinear or irregular rather than rectilinear or regular; (b) artificial features are absent or concealed; and (c) the presented landscape is dominated by vegetation, water, and mountains. However, previous studies did not pay attention to individual differences in immersion (e.g., Li and Sullivan, 2016; Lanki et al., 2017; Hicks et al., 2020), which is mentioned by Berto (2020). It may lead to different results in studies that used the same experimental protocol (Berto, 2005; Neilson et al., 2020).

Skilled performance highly relies on cognitive functions (Scharfen and Memmert, 2019), such as inhibition, working memory, flexibility, reasoning, and planning. For example, players must process information (e.g., working memory) to find the best solution with a time constraint (e.g., planning, reasoning, and creativity) to make a decision (Sakamoto et al., 2018). This process demands directed attention to block out irrelevant distractions and only focus on the important information (e.g., the movements of opponents and teammates). Since nature scenes could restore directed attention, there is an association between nature exposure and skilled performance shown in the conceptual framework.

Therefore, to apply this framework, future studies may consider about the following hypotheses: (i) nature exposure intervention could significantly improve skilled performance (technical and decision-making skills) in mentally fatigued athletes; (ii) the dosage or duration of the intervention plays a key role when apply the intervention. That is, longer and shorter duration may have different effects to influence skilled performance in mentally fatigued athletes, when nature stimuli (e.g., scenes) fixed. (iii) Voluntary attention (directed attention) may benefit different orders (high: working memory and flexibility vs. low: inhibition) of self-regulation in mentally fatigued athletes.

It is worthy to note that although the resource model is the best-known model of self-regulation so far, it may be manipulated to counter prior mental exertion (e.g., mental fatigue and ego depletion) (Sun et al., 2022b). However, it has met many challenges in the recent decade (Inzlicht and Schmeichel, 2012; Inzlicht et al., 2014). For example, glucose hypothesis. A study by Gailliot et al. (2007) showed that blood glucose could be the metaphorical resource. Despite its obvious appeal, this hypothesis was never test successfully (Finley et al., 2019).

In contrast the process self-regulation model recognized some shifted centres (e.g., motivation) of self-regulation and ignored the metaphorical resource (Inzlicht and Schmeichel, 2012; Inzlicht et al., 2014). However, Baumeister and Vohs (2003); Baumeister and Vohs (2007) updated the Strength Model and acknowledged the remedied motivation as an variable. Specifically, the authors contented that the monitoring process may compensate for relatively low self-regulatory resources through if individuals have high motivation to need standards.

## Limitations and future studies

It is important to highlight the limitations of the current review. Firstly, other factors that may affect cognitive performance were not discussed, such as motivation. Thus, the future studies may it to be a covariate, for motivation could determine the maximal level of effort and utilization of self-regulatory resources to perform to succeed in some tasks such as the Stroop task (Soutschek et al., 2014), and the subsequent skilled performance (Barte et al., 2018).

Secondly, the level of proficiency of athletes (elite, professional or amateur) was not discussed. However, Martin et al. (2018) indicated that professional athletes may have a better capacity of self-regulation than their recreational counterparts. Moreover, different types of athletes have their own traits in the execution of skilled performance such as open or close skills, thus they may have different responses regarding mental fatigue (Coyne et al., 2021). Future studies could consider the level of athletes and different types of sports when applying the conceptual framework.

Finally, the specific or practical duration was not discussed. Sun et al. (2022a) showed that 12.50 min may be the optimal choice for nature exposure. However, some studies showed that long time (e.g., 30 min) screen exposure could induce fatigue (Fortes et al., 2021a,b). That is, players may be fatigued again, if natural stimuli (e.g., scene) are presented for too long time on the screen, the relationship being inverted U-shaped. Future studies may examine more on the duration to find the cut-off point.

## Conclusion

Three theories (the psychobiological model of exercise performance, the strength model of self-regulation and attention restoration theory) could be integrated to generate a conceptual framework. The framework provided an overall picture of the counteractive effect of nature exposure to improve skilled performance, including technical and decision-making skills in mentally fatigued athletes. The improvement could be from the restoration of directed attention and self-regulation. However, some criteria should be paid attention to, such as the development of stimuli (e.g., nature scenes) and the duration of the intervention, and different traits of each individual (e.g., immersive tendency).

## Author contributions

All authors listed have made a substantial, direct, and intellectual contribution to the work, and approved it for publication.
