# Supplementary material for: Nature exposure might be the intervention to improve the self-regulation and skilled performance in mentally fatigue athletes: A narrative review and conceptual framework
Source: Front Psychol. 2022 Aug 2;13:941299. doi: 10.3389/fpsyg.2022.941299 (PMC9378859; doi:10.3389/fpsyg.2022.941299)

Table S1 - Detailed search strategy (series 1)

Database	Keywords	Results
PubMed<br>(Inception – March 2022)	(("mental fatigue" OR "mental exertion" OR "cognitive fatigue" OR "cognitive exertion" OR "mental exhaustion" OR "mental tiredness" OR “ego depletion”) AND ("athletic performance" OR "technical skill*" OR "skill*" OR "technique" OR "decision making" OR "performance")) AND (sport*)	49
Web of Science<br>(Inception – March 2022)	Ts = (“mental fatigue” OR “mental exertion” OR “cognitive fatigue” OR “cognitive exertion” OR “mental exhaustion” OR “mental tiredness” OR “ego depletion”) AND Ts = (“athletic performance” OR “technical skill*” OR “skill*” OR “technique” OR “decision making” OR “performance”) AND Ts = (sport*)	219
EBSCOhost<br>(Inception - March 2022)<br><br>(CENTRAL, SPORTDicus)	(“mental fatigue” OR “mental exertion” OR “cognitive fatigue” OR “cognitive exertion” OR “mental exhaustion” OR “mental tiredness” OR “ego depletion”) AND (“athletic performance” OR “technical skill*” OR “skill*” OR “technique” OR “decision making” OR “performance”) AND sport*	531
Scopus<br>(Inception – March 2022)	TITLE-ABS-KEY ("mental fatigue" OR "mental exertion" OR "cognitive fatigue" OR "cognitive exertion" OR "mental exhaustion" OR "mental tiredness" OR "ego depletion" AND "athletic performance" OR "technical skill*" OR "skill*" OR "technique" OR "decision making" OR "performance" AND “sport*”)	167

Table S2 - Detailed search strategy (series 2)

Database	Keywords	Results
PubMed<br>(Inception – March 2022)	(("mental fatigue" OR "mental exertion" OR "cognitive fatigue" OR "cognitive exertion" OR "mental exhaustion" OR "mental tiredness" OR “ego depletion”) AND ("athletic performance" OR "technical skill*" OR "skill*" OR "technique" OR "decision making" OR "performance")) AND (sport*)	26
Web of Science<br>(Inception – March 2022)	Ts = (“mental fatigue” OR “mental exertion” OR “cognitive fatigue” OR “cognitive exertion” OR “mental exhaustion” OR “mental tiredness” OR “ego depletion”) AND Ts = (“natur*”)	352
EBSCOhost<br>(Inception - March 2022)<br><br>(CENTRAL, SPORTDicus)	("mental fatigue" OR "mental exertion" OR "cognitive fatigue" OR "cognitive exertion" OR "mental exhaustion" OR "mental tiredness" OR “ego depletion”) AND (Natur*)	698
Scopus<br>(Inception – March 2022)	TITLE-ABS-KEY("mental fatigue" OR "mental exertion" OR "cognitive fatigue" OR "cognitive exertion" OR "mental exhaustion" OR "mental tiredness" OR “ego depletion” AND “natur*”)	311

Figure S1 – Summary of literature searching procedure

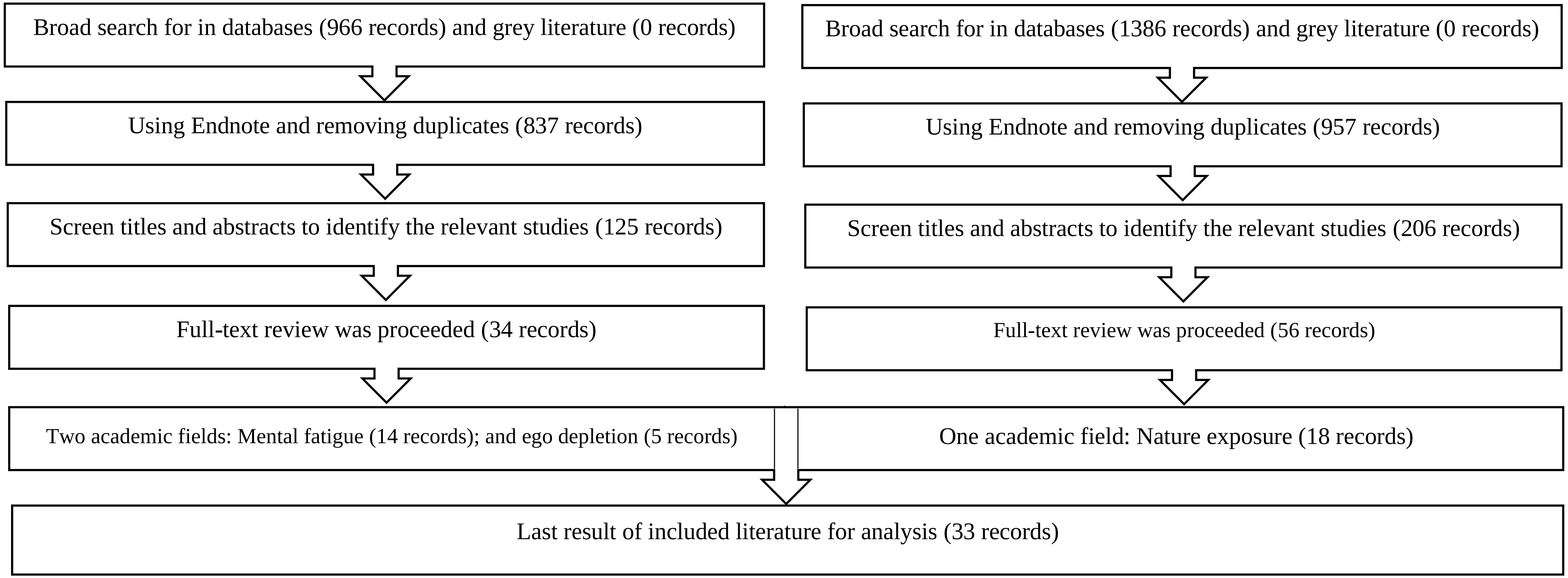

Supplement: Supplementary file 1 [file Data_Sheet_1.PDF]
